# Comfort in big numbers: Does over-estimation of doping prevalence in others indicate self-involvement?

**DOI:** 10.1186/1745-6673-3-19

**Published:** 2008-09-05

**Authors:** Andrea Petróczi, Jason Mazanov, Tamás Nepusz, Susan H Backhouse, Declan P Naughton

**Affiliations:** 1 Kingston University, Faculty of Science, School of Life Sciences, Penrhyn Road, Kingston upon Thames, Surrey, KT1 2EE, UK; 2 The University of Sheffield, Department of Psychology, Western Bank, Sheffield, S10 2TN, UK; 3 School of Business, UNSW@ADFA, Australia; 4 Budapest University of Technology and Economics, Department of Measurement and Information Systems, Hungary; 5 Carnegie Faculty of Sport and Education, Leeds Metropolitan University, Leeds, UK

## Abstract

**Background:**

The 'False Consensus Effect' (FCE), by which people perceive their own actions as relatively common behaviour, might be exploited to gauge whether a person engages in controversial behaviour, such as performance enhancing drug (PED) use.

**Hypothesis:**

It is assumed that people's own behaviour, owing to the FCE, affects their estimation of the prevalence of that behaviour. It is further hypothesised that a person's estimate of PED population use is a reliable indicator of the doping behaviour of that person, in lieu of self-reports.

**Testing the hypothesis:**

Over- or underestimation is calculated from investigating known groups (i.e. users vs. non-users), using a short questionnaire, and a known prevalence rate from official reports or sample evidence. It is proposed that sample evidence from self-reported behaviour should be verified using objective biochemical analyses.

In order to find proofs of concept for the existence of false consensus, a pilot study was conducted. Data were collected among competitive UK student-athletes (n = 124) using a web-based anonymous questionnaire. User (n = 9) vs. non-user (n = 76) groups were established using self-reported information on doping use and intention to use PEDs in hypothetical situations. Observed differences in the mean estimation of doping made by the user group exceeded the estimation made by the non-user group (35.11% vs. 15.34% for general doping and 34.25% vs. 26.30% in hypothetical situations, respectively), thus providing preliminary evidence in support of the FCE concept in relation to doping.

**Implications of the hypothesis:**

The presence of the FCE in estimating doping prevalence or behaviour in others suggests that the FCE based approach may be an avenue for developing an indirect self-report mechanism for PED use behaviour. The method may be successfully adapted to the estimation of prevalence of behaviours where direct self-reports are assumed to be distorted by socially desirable responding. Thus this method can enhance available information on socially undesirable, health compromising behaviour (i.e. PED use) for policy makers and healthcare professionals. The importance of the method lies in its usefulness in epidemiological studies, not in individual assessments.

## Background

The development of an epidemiology of performance enhancing drug (PED) use in sport has been restricted by the absence of a reliable and valid indicator of drug use [1,2]. Where conventional drug testing indicates prevalence around 2% [3], estimates from "how many people do you know who use PED" indicate the prevalence to be 6% [4] and in anecdotal reports up to 95% [5]. The absence of a reliable indicator has significant implications for assessing the value of interventions to ameliorate or eliminate drug use in sport. There is, however, the potential to develop a self-report measure using a known bias in human perceptions of social behaviour, the False Consensus Effect (FCE).

As noted above, prevalence estimates for PED use in sport range from 2% to 95%. Such a wide range of estimates indicates that there is poor evidence about the actual prevalence rate. That is, there is no reliable epidemiology of PED use among athlete populations [1]. Laure [6] reports an attempt to develop an epidemiology of androgenic-anabolic steroid (AAS) use in France, suggesting that 10–20% of athletes use such substances regardless of age, sex or sport. A comprehensive study across six European countries that relied upon self reports among university students indicated that 2.6% were willing to admit use of PED [7].

The failure to generate an acceptable epidemiology is predicated based upon the methods used to detect the prevalence of PED use in sport being flawed. The administrative, financial and scientific constraints of biomedical testing have become received wisdom with acknowledgement of the drugs in sport 'arms race' between new drugs and detection technologies. That is, biomedical detection is unlikely to give an accurate indication of prevalence due to the combination of an inability to test universally and the introduction of drugs undetectable by contemporary methods [8].

The application of typical social science methods to generating estimates of prevalence leads to problems such as the reliability of self report, non-response bias or social desirability [2]. Who is asking the question may also contaminate the response; it would be a brave athlete who admitted to PED use on a survey run or sponsored by a National Anti-Doping Organisation. Likewise, it would be scientifically invalid to infer anything about substance use behaviour from stated attitudes or intentions towards PED use given the tenuous relationship between the two.

With the failure of typical biomedical or social science approaches to provide a basis for developing an epidemiology of PED use in sport, atypical approaches are called for. One such atypical technique called the 'Random Response Technique' (RRT) has proven to be more reliable when sensitive issues such as abortion, illicit drug use, opinion about capital punishment or shoplifting are investigated [9-11]. Using the RRT, Simon and colleagues recently showed a comparatively high (12.5%) prevalence of doping use among gym users [12].

The aim of this paper is to propose an alternative indirect approach which has been used in sociology but is new to doping research and relies on social projection. The notion of social projection was introduced more than 80 years ago [13] and the method has been extensively used in social psychology [14-19]. The false consensus effect arose from psychology's efforts to explain discrepancies in social judgement. Specifically, the effect describes the considerable overestimation of behaviour in which a person engages, and a slight underestimation of behaviour absent from a person's repertoire [18]. That is, over-estimating a particular behaviour indicates that the person who makes the estimate (and overestimates the behaviour) is likely to be engaged in the same act. Research regarding attributive projection (the tendency of people to project their own characteristics onto others) [20], the FCE and uniqueness bias have been particularly pervasive in social psychology [18]. According to the FCE theory [21], individuals often tend to overestimate the extent to which others behave the same way as they do, especially if the behaviour in question is deemed to be socially questionable or unacceptable. This phenomenon is explained by a part motivational, part cognitive process resulting in people believing that their own action is a relatively common behaviour. The effect appears to be present even when objective statistics and information on the bias effect are provided, indicating the intractable and egocentric nature of this biased social perception [22].

For example, self reporting marijuana smokers overestimated the proportion of users in the general population by 28% whereas non-smokers of marijuana overestimated the rate of use by 14% [18]. The directions of these estimations were congruent with the self-reported behaviours (i.e. non-users under-estimated and users over-estimated) in a study regarding students' use of amphetamines. In this report students who abstained from amphetamines typically underestimated (estimate 29% *versus *35% reported) and users overestimated (estimate 48%) prevalence of amphetamine use but not other behaviour, suggesting that this FCE is behaviour-specific and does not generalise to other similarly ostracised acts [19].

Recent marketing research investigating consumer behaviour demonstrated that overestimation is greater when an individual holds positive feelings toward the subject [23]. In addition to finding further evidence for the FCE, Monin & Norton [17] also demonstrated the existence of a strategy people use to justify their undesirable behaviour. This strategy typically involves justification based on the sense of comfort in large numbers (i.e. many are doing so) or citing special mitigating circumstances. It was also shown that bias estimation (whether over or under-estimation) is rooted in the social perception of the behaviour, not in the behaviour itself [17]. The estimation of others' behaviour was influenced by the combination of two conditions: i) the person's own behaviour and, ii) what was desirable in the given situation. As such, estimation bias may change over time as one or both of these conditions change.

Whilst its causality has remained unknown, the relationship between self-involvement and overestimation has been repeatedly evidenced with regard to smoking, drinking and illicit drug use [24-26]. It has been suggested that perceived prevalence may act as a normatively prescribed behaviour [24] and actually initiates the behaviour. For example, if emerging athletes believed that using PEDs is necessary to be successful in high performance sport and that everyone uses PEDs, this belief may work as a perceived norm for these athletes and motivates them to do as the others and start taking PEDs. While it is a plausible application of the FCE, its validity requires further evidence, preferably from longitudinal studies. For the purpose of the present proposal, it is sufficient to assume that significant overestimation signals involvement, namely doping use or intention to use.

Estimation of prevalence has also appeared in doping research. Pearson & Hansen's study of athletes at the 1992 Winter Olympics provides an insight into how the FCE might work in an anti-doping context [27]. In this study, athletes were asked to estimate the prevalence of doping or certain PEDs among their peers. For example, where the reported positive cases vary around 2% [3], 67 of 155 athletes (43%) surveyed by Pearson & Hansen thought that more than 10% of athletes in their sports used anabolic steroids, and a further 53 (34%) gave an estimate between 1% and 9% [27]. A survey conducted among Finnish Olympic athletes revealed similar results. Whilst none admitted using PEDs, 42.5% from stress power sports and 37.0% of endurance athletes reported that they personally know another athlete who uses PEDs [28].

In the context of a review for WADA, Backhouse and colleagues report that unvalidated self-reported PED use among elite athletes typically ranges between 1.2% and 8% [29]. Conversely, projective techniques where athletes are asked to estimate how many team mates or competitors used PED, the estimate increased to between 6% and 34%. This divergence in estimates appears large for random sampling differences and may be better explained by the FCE. Using FCE-based surveys may equip researchers, policy makers and health care professionals with a more realistic estimate of PED use by the athlete population. It is envisaged that in its broader aspects, this study would help to provide guidance for the general population with respect to PED use, particularly for non-prescription anabolic steroids, amphetamines and/or analgesics.

## The hypothesis

Applying the FCE concept to PEDs in a sport context, it is hypothesised that athletes who use PEDs overestimate prevalence of doping in their sport and in sport more broadly, compared to non-users. The measurement tool we propose to develop for doping prevalence estimation is based on the FCE, assuming that the effect is present for illicit or banned drug use. What differentiates the proposed approach from reported projected use is how the estimation made by respondents is used. Typically estimates are reported at face value and discussed as prevalence in the population. We propose to use estimates to gain information about the *individual *who makes the estimates and *not the population *for which the estimates are made. While there are no epidemiology data for drugs in sport against which to compare athlete responses [1], it is the magnitude of over- or underestimation that may provide the indicator. The indirect nature of asking athletes about prevalence may yield an indicator suitable for epidemiological and social science based research to begin cross-sectional descriptive or prospective causal models of athlete PED use.

## Testing the hypothesis

Determining the level of over- or underestimation will be conducted by calculating deviation from the publicly established prevalence rate of 2% [3] and the prevalence rate calculated from the presence of doping in the sample (users/non-users). Estimates can be solicited in various forms ranging from direct questions (i.e. 'In your opinion, what percentage of others in your sport use PEDs?' or 'To your knowledge, what proportion (%) of your fellow athletes use PEDs?') to hypothetical scenarios (i.e. 'Under circumstances X, what percentage of the athletes would use PEDs?'), where depending on the research question, using different hypothetical situations can be used as experimental manipulation. Estimates made by user and non-user groups will be compared and the differences tested for statistical significance:

H1: μ_1 _> μ_2_,

H2: (μ_1 _- P) > (μ_2 _- P)

where μ_1 _and μ_2 _denote population estimate for users and non-users, respectively and P is the doping prevalence in the population.

Significantly higher estimates made by the user group will provide empirical evidence for the FCE. Part of this test for association includes developing an estimate for confidence in the level of overestimation and their corresponding odds ratios (OR). OR is defined as the ratio of the odds of doping use occurring in one group (high prevalence estimators) to the odds of it occurring in another group (lower prevalence estimators), or to a sample-based estimate of that ratio. The calculation of the odds ratio will be based on Fisher's Exact Test (FET). The advantage of the FET over a simple calculation of the odds ratio is that FET provides a confidence interval for the odds ratio.

An OR of 1 indicates that doping use is equally likely in both high- and low estimating groups. An OR greater than 1 indicates that doping is more likely (may be many times) in the high estimators group, whereas an OR below 1 indicates that doping is less likely in this group in comparison to the other, low estimators group. Owing to the phenomenon that OR sometimes overstates relative positions, it is proposed that the log OR value will be used.

## Proposed approach to testing the hypothesis

Social psychology research on the FCE typically uses self-reported data to create the two fundamental groups: those who are involved in the investigated behaviour and those who are not involved. In these cases, self reports on behaviour were taken at face value and treated as a truthful and accurate report on one's behaviour. Assuming that such self reports, especially in regards to controversial behaviour, are free of response bias is naïve and self-reported information (when possible) should be verified with or replaced by objective measures at least during the pilot study phase. For example, such objective information can be obtained via biochemical analyses. Hair analyses, in particular, provide a non-invasive approach to the simultaneous assessment of multiple metal ions used in mineral supplements, steroids [30] and many social drugs (cannabis, amphetamines, opiates and cocaine) that are prohibited during competition. Hair analysis also has the advantage of having a considerably longer detection window that allows testing for habitual use (chronic and past consumption) of drugs [31]. Therefore, it is proposed that self-reports will be corroborated with biochemical analysis. More precisely, hair assays will be used to detect the use of steroids, selected social drugs and multivitamins/iron supplements as control measures. The biochemical validation is then used to verify whether over- or underestimation is associated with use or abstinence and odd ratios will be calculated based on the magnitude of over-estimation by the user groups.

In cases when biochemical analyses for the entire sample is not feasible (i.e. owing to large sample sizes in epidemiological studies), it is suggested that biochemical analysis should be used on a small *representative *sample prior to or as part of the main data collection. This would provide guidance as to what degree self-reports are distorted (most likely under-reported) and information to be used to adjust self-reports from large scale studies accordingly.

In recent years, rigorous methodologies have been developed and validated for the assessment of exposure to a wide range of supplements, drugs and toxins, including metal ions [32-35]. Hair samples should be untreated hair cuts from close to the scalp, typically at the posterior vertex, although pubic, axillary, arm, chest or thigh hair can also be used with adjusted cut off values [31]. The samples should be stored in paper envelopes or folded scaled paper with ends fixed and marked if timescale was an issue. Scalp hair normally grows approximately 100 mm in every 30 days, therefore time or frequency of the drug consumption can also be detected within the generally accepted 90 days detection window.

For drug analyses, the hair samples are sectioned (>1 mm) and stirred in methanol for 167 hours at 40°C prior to evaporation [31]. Samples for metal ion analyses are solubilised by heating at 150°C for 30 min after adding a 2:1 HNO_3_: H_2_O_2 _mixture [33]. Methods have been developed for analysis of metals, social and performance enhancing drugs using inductively-coupled mass spectrometry (ICP-MS), gas chromatography-tandem mass spectrometry (GC-MS/MS) and liquid chromatography-tandem mass spectrometry (LC-MS/MS).

## Proof of concept: a pilot study

To establish the presence of the FCE in relation to doping, a small scale pilot study was conducted. The primary aim of this pilot study was to provide proof that the FCE is present in the perception of doping behaviour. In addition, the study also served as validation of the measurement tool (questionnaire) designed to obtain self-reported information from the athletes.

### Methods

To investigate whether a relationship exists between doping use and potential doping use and estimation of others' use and potential use, a questionnaire was developed containing questions of the following: i) self-reported doping use (recorded as Y/N), ii) estimated doping use of others (as %) and eight hypothetical scenarios of doping use forming the Hypothetical Doping Scenarios (HDS). For estimating potential doping behaviour of others, respondents were asked to estimate the proportion (as %) of others who would use doping. Respondents were also asked to report whether or not they would use doping in a prescribed situation (HDS-Self, recorded as Y/N). For the questions, see Additional File 1: Direct doping estimate of others, self reported doping behaviour, HDS and HDS-Self. The questions were preceded by a classification and brief definition of the drugs (see Additional file 2: Definition of nutritional supplements and doping. In congruence with the WADA regulation, there was no distintction made between social drugs and other substances if they were used for performance anhenacing purposes.

### Analyses

Self reports were used to establish the user categories. The HDS-Self score was used to group participants as users vs. non-users, where athletes with HDS-Self ≥ 1 were classified as potential user. Direct self report had binary values (No = 0, Yes = 1). For the purpose of the analyses, only those athletes were considered doping users who were classified 'user' in both categories (direct report and hypothetical use). Similarly, non-user athletes were those who were classified as 'non-users' in both categories. Owing to the ambiguity in the other two categories that will require further investigation, 39 athletes who fell in these two categories were excluded from the comparison of population estimates. Categorisation for nutritional supplement users was conducted in the same manner.

Population estimates for doping and nutritional supplement use were obtained in two forms. Athletes were asked in a straightforward manner to estimate the percentage of athletes, in general, who use doping or nutritional supplements. Hypothetical situations identical to the self-reported hypothetical situations (HDS-Self) were also used. Estimates given as percentages were used as reported for the direct general estimates and were averaged for the eight scenarios. Comparisons of group means were performed with Mann-Whitney non-parametric statistics using SPSS 15.0, and R statistical software was used for Fisher's Exact Test for Count Data.

### Sample

Data were collected among UK sports science students and student athletes (n = 142) using a web-based anonymous questionnaire. 124 participants met the criteria of taking part in sport at the designated competitive level. Competitive level was defined as regular participation in organised sports competition. Given the nature of the present sample (sports science students and student athletes), competition equates club level competition here. The sample consisted of 46 (37.1%) female and 78 (62.9%) male athletes with mean age of 21.47 ± 5.53. User vs. non-user groups were established using self-reported information on doping use and intention to use PEDs in hypothetical situations. Based on the self reported doping use and potential use, respondents were categorised into four groups: users with current and potential use (n = 9), potential users with no current use (n = 31), 'ambiguous' users with current use but denied potential use (n = 8) and non-users (n = 76).

### Results and discussion

**Figure 1:**
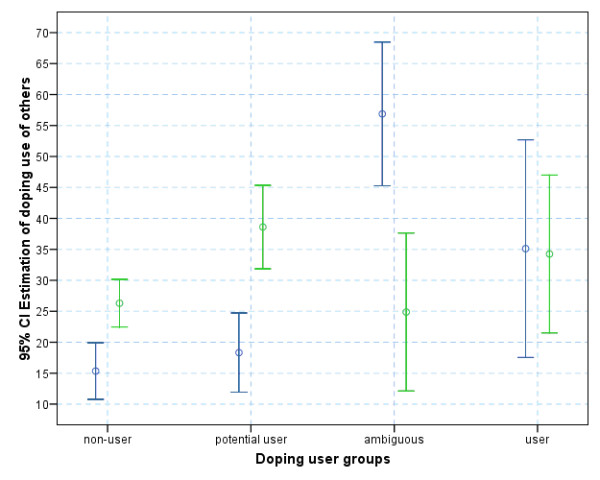
Estimation of doping use (blue) and hypothetical doping use (green) among others (displayed as means and 95% confidence intervals).

Scale reliability coefficients for HDS scales were reassuringly above the customary cut-off value (α = .886 for PED and α = .917 for NS), suggesting good internal consistency. Observed differences in the mean estimation of PED use made by the user group exceeded the estimation made by the non-users (35.11% vs. 15.34% for general doping and 34.25% vs. 26.30% in hypothetical situations, respectively) providing evidence in support of the FCE concept (Figure 1). The difference, however, was only statistically significant for the general estimation (U = 143.00, p = .004) but not for the summarised hypothetical situations (U = 247.00, p = .175, d = .476). The other two groups (potential users and the ambiguous group) showed considerable inconsistency, suggesting that these answers (as well as the self-reported information on which group membership was established) have most likely been influenced by the perceived need for socially desirable responding. Notably, the variance in estimations was considerably less among the self-declared clean athletes.

Following the methods used in previous research [19,25], the accuracy of estimates were calculated as the difference between the estimate given by the participants (X) and the actual population figure (P). The population figures we used were i) the official rate of positive doping tests reported yearly by the WADA (2%) and ii) self-reported doping behaviour in the sample (13.7%, 95%CI = .08, 20.0). The accuracy of an estimate is the degree to which responses reflect reality. Accuracy of the estimates for our sample using i) self-reported information for population prevalence and ii) official rate of positive tests showed significant difference between users and non-users (U = 143.00, p = .004 and U = 143.00, p = .004, respectively). However, the problem with this method arises from the uncertainty regarding population prevalence. The prevalence rate calculated from self-reports (which itself may be under-reported owing to the social desirability effect) suggests a considerably higher prevalence rate compared to the official yearly reports of the World Anti-Doping Agency (13.7% vs. 2%).

Notably, even the lowest estimations given by athletes were considerably above the average rate of positive doping tests (ca 2% of all tests according to the WADA yearly reports [3]), which may either signal the widespread belief that competitors are using doping (hence it is perceived as normative behaviour) or give a closer and more realistic estimate of doping prevalence. More importantly, the significant overestimation by doping users suggests that if such an indirect method is further refined and validated, it may be successfully employed in large scale prevalence studies as a low-cost, reliable measurement tool to capture the prevalence of doping behaviour.

From the FET, the odds ratio is 6.025, 95%CI = 1.365, 31.186 (ln = 1.82), suggesting that doping use is more likely among those who estimate doping use in others beyond the sample prevalence upper 95%CI (20%). Notably, the lower bound of the 95% CI is above 1, suggesting that the difference is significant at the 95% confidence level. The p value of .007 provides further reassurance that the true OR is > 1.

Results regarding nutritional supplements suggest that social projection is influenced by the social judgement of the behaviour. For nutritional supplements (NS), 57 athletes (46%) reported current use with a further 61 who would consider using NS and 6 athletes rejected NS use under any circumstances. Unlike PED, the 'ambiguous' cell (current use with denied hypothetical use) was empty for NS.

The comparison using estimated NS prevalence as outcome revealed similar but less marked patterns than the same analyses with projected PED use. Doping users' estimation of NS use of others were higher than the estimation made by non-users for both general estimation (54.15 ± 30.19 vs.46.72 ± 27.34%) and hypothetical situations (74.79 ± 22.90% vs. 59.68 ± 20.40%), but the differences were not or close not non-significant (U = 295.00, p = .500 and U = 203.50, p = .048, respectively). The mean direct prevalence estimations (54% and 47%) were close to the actual sample prevalence of 46%. The estimates of hypothetical NS use by others (73% vs. 60%) were actually below the actual self-reports of the same behaviour (95%).

## Conclusion

It is evident from the literature that categorisation (involved vs. not involved in an act) was typically based on self-reports, which are known to be susceptible to response bias. Results from this pilot study, in addition to providing important evidence for the presence of the FCE, have flagged this problem as well. Social projection appears to be dependent on the social judgement of the behaviour. Therefore, it is suggested that FCE-based assessment, coupled with using objective indicators of behaviour (i.e. biochemical analyses) should be used in prevalence studies on socially sensitive issues (such as using PEDs), instead of relying on the dubious results of self-reports.

## Significance

Epidemiological and social science based research into drugs in sport have been restricted by the absence of a viable dependent variable upon which to differentiate users from non-users. Existing self-report measures are assumed to be significantly under-reported given that people are unlikely to incriminate themselves by admitting use and unable to provide a sound basis for policy makers. Thus, alternative methods of inquiring about performance enhancing substance (or method) use are needed. One such alternative method makes use of results from social psychology to develop a possible proxy that may prove reliable, benefiting from the FCE. The measurement tool is not envisaged to be used to gather data on projected use, but rather, employed as an implicit self-report method. A model will be developed to give an estimation of 'own' use based on the projected use.

Biochemical validation of self-reported drug use can provide researchers with objective information upon which categorisation (users vs. non-users) is made, hence it is proposed to be used for validation of self-reports. Therefore this project proposes an elegant integration of biochemistry, social psychology and statistics to tackle the problem of obtaining reliable estimate for the prevalence of doping.

The measurement tool is to be used as a research tool to gather information on prevalence of PED use but it is not intended to be a diagnostic tool for individual assessment. The method may also be successfully adapted to the estimation of prevalence of behaviours where direct self-reports are assumed to be distorted by socially desirable responding. Thus this method is designed to enable collecting reliable information regarding the prevalence of PED use; and to enhance health care professionals' understanding of PED use. Ideally, these studies, along with recent investigations into PED use in elite athletes [36,37] concerning rationale vs. practice, will inform health care professionals to target populations at risk of PED use. Planning effective anti-doping or anti-drug prevention requires accurate information reflecting the true scale of PED or drug use in various populations (i.e. athletes, non-athletes, adolescents, adults, elderly, etc). Owing to previously demonstrated difference in strength of the FCE [24,25,38,39], normative feedback type intervention might be especially effective among adolescents.

## Competing interests

The authors declare that they have no competing interests.

## Authors' contributions

AP formulated the testable hypothesis, developed the research design, developed the questionnaire, collected and analysed the pilot data and drafted the manuscript. DPN assisted in formulating the testable hypothesis, developed the protocol for the biochemical testing and contributed to drafting the manuscript. JM initiated the project, contributed to developing the hypothesis and protocol and writing the manuscript. TN developed the web-based test site and prepared the data for statistical analyses. SHB assisted in developing the questionnaire and collected data for the pilot study. All authors have read and approved the final version of the manuscript.

## Supplementary Material

Additional File 1 Direct doping estimate of others, Self reported doping behaviour, HDS and HDS-Self. The file shows the questions used for self-reporting and estimating doping behaviour directly and in hypothetical situations. Click here for file

Additional file 2 Definition of nutritional supplements and doping. The file provides definitions for drug categories used in the questionnaire. Click here for file
