# Supplementary material for: Comfort in big numbers: Does over-estimation of doping prevalence in others indicate self-involvement?
Source: J Occup Med Toxicol. 2008 Sep 5;3:19. doi: 10.1186/1745-6673-3-19 (PMC2553062; doi:10.1186/1745-6673-3-19)
Supplement: Additional File 1 — Direct doping estimate of others, Self reported doping behaviour, HDS and HDS-Self. The file shows the questions used for self-reporting and estimating doping behaviour directly and in hypothetical situations. [file 1745-6673-3-19-S1.doc]

**Additional file 1: Direct doping estimate of others, Self reported doping behaviour, HDS and HDS-Self**

**Hypothetical Doping Scenarios for estimating the potential doping behaviour of others (HDS)**

| 1 | If a banned method was available and non-detectable, what % of others in your sport would use the method if they were sure that its use would lead to a win? |
| --- | --- |
|  |  |
| 2 | If a banned method was available and non-detectable what % of others in your sport would use the method if it was assumed to level the playing field and give a chance to win? |
|  |  |
| 3 | In preparation for a competition the coach suggests using a banned method to enhance athletic performance. What % of others in your sport would use the banned method in this instance? |
|  |  |
| 4 | In preparation for a competition a medical doctor suggests using a banned method to improve recovery rate. What % of others in your sport would use the banned method in this instance? |
|  |  |
| 5 | In preparation for a competition a family member suggests that you using a banned method to enhance athletic performance. What % of others in your sport would use the banned method in this instance? |
|  |  |
| 6 | In preparation for a competition, a fellow athlete suggests that using a banned method to enhance athletic performance. What % of others in your sport would use the banned method in this instance? |
|  |  |
| 7 | If a banned method was available, effective and non-detectable, what % of others in your sport would take the substance if there were only minor side effects associated with its use? |
|  |  |
| 8 | If a banned method was available, effective and non-detectable, what % of others in your sport would use the banned method in this instance if there were major side effects associated with its use? |

**Hypothetical Doping Scenarios for self-estimation of the potential doping behaviour (HDS-Self)**

| 1 | If a performance enhancing method was available and non-detectable would you use it if it was a banned method but its use would lead to a win? |
| --- | --- |
|  |  |
| 2 | If a performance enhancing method was available and non-detectable would you use the method if you thought that its use would level the playing field for you to win? |
|  |  |
| 3 | You are preparing for a competition and your coach suggests that you use a performance enhancing method to enhance your performance. Would you do it if it was a banned method? |
|  |  |
| 4 | You are preparing for a competition and your medical doctor suggests you use a performance enhancing method to improve your recovery rate. Would you do it if it was a banned method? |
|  |  |
| 5 | You are preparing for a competition and your family member suggests that you use a banned method to enhance your performance. Would you do it if it was a banned method? |
|  |  |
| 6 | You are preparing for a competition and one of your fellow athletes suggests that you use a method to enhance your performance. Would you do it if it was a banned method? |
|  |  |
| 7 | If a method was available, effective and non-detectable with only minor side effects associated with its use. Would you do it if it was a banned method? |
|  |  |
| 8 | If a method was available, effective and non-detectable but with major side effects associated with its use. Would you do it if it was a banned method? |

**Direct estimation** of others’ perceived doping behaviour:

| What % of others in your sport has used a banned substance? |
| --- |

**Self-reported doping behaviour:**

| Have you ever used a banned substance? |  |
| --- | --- |
|  |  |

Note: The same set of questions were asked with nutritional supplements.
