# Supplementary material for: Comfort in big numbers: Does over-estimation of doping prevalence in others indicate self-involvement?
Source: J Occup Med Toxicol. 2008 Sep 5;3:19. doi: 10.1186/1745-6673-3-19 (PMC2553062; doi:10.1186/1745-6673-3-19)
Supplement: Additional file 2 — Definition of nutritional supplements and doping. The file provides definitions for drug categories used in the questionnaire. [file 1745-6673-3-19-S2.doc]

**Additional file 2: Definition of nutritional supplements and doping**

For the purpose of this survey, unless specified,

- **‘nutritional supplements’** are vitamins (e.g. vitamin A, B, C, E, D, etc.), minerals (e.g. iron, zinc, calcium, magnesium, etc.) and non-vitamin non-mineral substances including herbals and botanicals (e.g. creatine preparations, St John’s worth, Ginco biloba, Kava kava, Echinacea, Ma huang, Guarana, protein solutions, glucosamine, coenzyme Q10, lecithin, melatonin, fish oil, ginseng, shark cartilage, etc.).
- **‘doping’ or ‘banned substances’** are those substances that are prohibited by the World Anti-Doping Agency or other governing body in training and/or competition.
